# Derepression of specific miRNA-target genes in rice using CRISPR/Cas9

**DOI:** 10.1093/jxb/erab336

**Published:** 2021-07-20

**Authors:** Yarong Lin, Yiwang Zhu, Yuchao Cui, Rui Chen, Zaijie Chen, Gang Li, Meiying Fan, Jianmin Chen, Yan Li, Xinrui Guo, Xijun Zheng, Liang Chen, Feng Wang

**Affiliations:** 1 Institute of Biotechnology, Fujian Academy of Agricultural Sciences/Fujian Provincial Key Laboratory of Genetic Engineering for Agriculture, Fuzhou, China; 2 Xiamen Key Laboratory for Plant Genetics, School of Life Sciences, Xiamen University, Xiamen, China; 3 Shenzhen Branch, Guangdong Laboratory of Lingnan Modern Agriculture, Genome Analysis Laboratory of the Ministry of Agriculture and Rural Affairs, Agricultural Genomics Institute at Shenzhen, Chinese Academy of Agricultural Sciences, Shenzhen, China; 4 Oklahoma State University, USA

**Keywords:** CRISPR/Cas9, de-repression, in-frame, miRNAs, rice

## Abstract

MicroRNAs (miRNAs) target specific mRNA molecules based on sequence complementarity for their degradation or repression of translation, thereby regulating various developmental and physiological processes in eukaryotic organisms. Expressing the target mimicry (MIM) and short tandem target mimicry (STTM) can block endogenous activity of mature miRNAs and eliminate the inhibition of their target genes, resulting in phenotypic changes due to higher expression of the target genes. Here, we report a strategy to achieve derepression of interested miRNA-target genes through CRISPR/Cas9-based generation of in-frame mutants within the miRNA-complementary sequence of the target gene. We show that two rice genes, *OsGRF4* (*GROWTH REGULATING FACTOR* 4) and *OsGRF8* carrying in-frame mutants with disruption of the miR396 recognition sites, escape from miR396-mediated post-transcriptional silencing, resulting in enlarged grain size and increase in brown planthopper (BPH) resistance, in their respective transgenic rice lines. These results demonstrate that CRISPR/Cas9-mediated disruption of miRNA target sites can be effectively employed to precisely derepress particular target genes of functional importance for trait improvement in plants.

## Teaser

CRISPR/Cas9 is used as a strategy to achieve derepression of miRNA-target genes to create elite traits in rice.

## Introduction

MicroRNAs (miRNAs) are a class of single-strand non-coding RNAs, of typically 20-24 nt in length (Jones-Rhoades *et al.*, 2006; Zhang *et al.*, 2006; Li *et al.*, 2017; Ghini *et al.*, 2018). They bind to mRNAs by specific complementary base-pairing mechanisms, and mediate post-transcriptional gene silencing. In plants, miRNAs have been shown to be involved in numerous biological processes, including growth, development, stress responses and hormone signalling, making miRNAs strong targets for trait improvement (Khraiwesh *et al.*, 2012; Tang and Chu, 2017). Thus, exploring the effective approaches to interrogate miRNA functions is necessary.

Target mimicry (MIM), consisting of a non-cleavable RNA that forms a non-productive interaction with a complementary miRNA, provides a powerful technology that can be used to inhibit the activity of miRNAs (Franco-Zorrilla *et al.*, 2007). Based on the MIM technology, short tandem target mimicry (STTM), which is composed of two short sequences mimicking small RNA target sites, linked by a short spacer, induces the degradation of specific miRNAs (Yan *et al.*, 2012; Zhao *et al.*, 2019). Moreover, several studies have shown that high efficiency mutagenesis of miRNA genes can be obtained by CRISPR/Cas9 technology (Miao *et al.*, 2020; Zhou *et al.*, 2017). Such miRNA-targeted approaches provide effective tools to interfere with endogenous miRNA activity. In addition, the transgenic expression of miRNA-resistant targets, where multiple synonymous mutations are introduced in the miRNA binding site, is employed to escape the repression of endogenous miRNAs (Li and Millar, 2013; Song *et al.*, 2013). Overall, multiple approaches have been developed to research on miRNA function and the miRNA-target regulatory module, providing guidelines for crop improvement.

miR396 is a miRNA that is conserved among the dicot and monocot plants. In Arabidopsis, miR396 targets a specific family of transcription factors, GROWTH-REGULATING FACTORS (GRFs), which are known to control cell proliferation during leaf development (Jones-Rhoades and Bartel, 2004; Liu *et al.*, 2009). miR396 has also been found to down-regulate the abundance of *GRFs* mRNA, and is implicated in the control of cell differentiation and organogenesis in Poaceae. Several studies in rice have identified a natural occurring allele of *OsGRF4*, a 2 bp missense mutation in the miR396 recognition site, perturbing repression of miR396 and increasing grain size (Duan *et al.*, 2015; Hu *et al.*, 2015; Che *et al.*, 2016; Chen *et al.*, 2019). Therefore, it is possible that we can introduce miR396-resistant targets into plants to destroy the inhibition by miR396 and improve specific agronomic traits.

Given that the CRISPR/Cas9 system can produce in-frame and frame-shift mutants (Shan *et al.*, 2013; Zhang *et al.*, 2014; Zhu *et al.*, 2019), we aimed to generate in-frame mutants in the miR396 recognition site of interested target genes, which would make the target genes escape from post-transcriptional inhibition, but also keep the function of target genes. To evaluate whether the strategy works, we selected *OsGRF4* and *OsGRF8*, which are regulated by miR396 to control grain size and brown planthopper (BPH) resistance, respectively (Dai *et al.*, 2019). Through the CRISPR/Cas9 system, we successfully destroyed the miR396-complementary sequence and produced in-frame *osgrf4* and *osgrf8* mutants in which the lengths of deletions or insertions were in multiples of three bases. Subsequently, the elevated expression of the in-frame *osgrf4* and *osgrf8* mutants led to enlarged grain size and increased BPH resistance respectively. Consequently, our results constitute a strategy for acquiring miRNA-resistant mutants using CRISPR/Cas9 that could be readily implemented in plants.

## Materials and methods

### Plant material and growth conditions

*Oryza sativa* L. *indica* ‘Shuhui143’ (‘S143’) was used as the wild type (WT) control and transformation host. The majority of the WT and transgenic plants were cultivated under a standard greenhouse at Fuzhou experimental station (26.08°N, 119.28°E), Fujian Province, China. The growing season began in May and extended to mid-October. Mature seeds were germinated in half-strength Murashige and Skoog (MS) medium in a 26 °C incubator for 14 d and then transferred to soil. The seedlings were grown under standard greenhouse conditions (14 h light at 30±1 °C/10 h dark at 25±1 °C).

### Vector construction and rice transformation

The single guide RNA (sgRNA) sequences targeting the flanking sequences of the miR396 complementary sequence in the *OsGRF4* and *OsGRF8* alleles were designed as predicted by the CRISPR-P program (Lei *et al.*, 2014). The oligonucleotide dimers of designed sgRNA sequence were cloned into a CRISPR/Cas9 plant expression vector VK005-01 (Viewsolid Biotech, Beijing, China) following the manufacturer’s specifications. The constructs carried a Cas9/sgRNA T-DNA and a designed sgRNA sequence, driven by the maize ubiquitin promoter and the rice U6 promoter, respectively.

The resulting constructs were confirmed by sequencing and then introduced into *Agrobacterium tumefaciens* strain EHA105. Next, the strains were transferred into rice calli of ‘S143’ by *A. tumefaciens*-mediated rice transformation, as described previously (Hiei *et al.*, 1994).

### Genotype analysis

Total genomic DNA from fresh leaves was extracted using the cetyltrimethylammonium bromide (CTAB) method (Stewart and Via, 1993). The *Cas9* gene was amplified to evaluated the presence/absence of the Cas9/sgRNA T-DNA. Using specific primers, transgenic samples were performed to amplify the sgRNA target region of *OsGRF* genes and subsequently subjected to Sanger sequencing. Sequencing results were decoded with an established tool, as described previously (Liu *et al.*, 2015). DNA sequences were aligned using Clustal Omega (Sievers *et al.*, 2011).

### Trait measurements

A total of 96 plants of T_2_ mutagenesis of *grf4-*#2, *grf4*-#25, *grf4*-#1, *grf4*-#14 and T_1_ mutants *grf4-2*-#7, *grf4-2*-#46 were grown for analysis. Grain length and grain width with 300 grains were measured when the grains were completely matured. A total of 1000 fully filled grains were chosen to investigate grain weight (1000-grain weight). Plant height, tiller number, primary branch number per panicle, secondary branch number per panicle, grain number per panicle and yield per plant were also measured and analyzed from the plant materials.

### RNA isolation and qRT-PCR analysis

Total RNA was extracted from rice young panicles and leaves using TRIZOL (Life technologies, Carlsbad, CA, USA). The cDNAs were reverse transcribed using the First Strand cDNA Synthesis Kit (Thermo, Waltham, MA, USA). Quantitative RT-PCR was performed with the SYBR Premix Ex Taq (Takara, Otsu, Shiga, Japan) as per the manufacturer’s instructions, and the rice *UBIQUITIN* and *OSACTIN1* genes were used as endogenous controls. For the detection of miR396, reverse transcription was conducted using stem-loop RT primers (Supplementary Table S1), and the *U6* gene was regarded as an internal reference in quantitative RT-PCR. Relative expression was analysed using the 2^-∆∆Ct^ method. Data from three biological replicates and three technical repetitions were collected, the mean value with SD was plotted for each individual genome editing event and the controls based on three biological replicates.

### Detection of brown planthopper resistance and determination of total flavonoids

Approximate 3-week-old seedlings were used for brown planthopper (BPH) resistance test. Each line of WT ‘S143’, in-frame and frame-shift *osgrf8* mutants was infested with 12 second-instar BPH nymphs with at least three replicates (Zhao *et al.*, 2016). A week later, the resistance level (susceptible or resistance) of plants were evaluated (Dai *et al.*, 2019).

Approximately 1 g milled powder from fresh leaves of 30-day-old seedling were extracted in 10 ml of 50% ethanol for 1 h with shaking and repeated three times. The extract was centrifuged at 12 000 × *g* for 10 min, and 0.5 ml of supernatant was diluted to 5 ml with 30% ethanol, After 5 min, 0.3 ml of 0.5 M NaNO_2_, 0.3 ml of 0.3 M AlCl_3_, and 2 ml of 1 M NaOH were added and the reaction was made up to 10 ml using distilled water. After 15 min of incubation, the absorbance at 510 nm was recorded against a blank sample. The total flavonoid content was calculated as the equivalents of Rutin used as the standard (Loyola *et al.*, 2016). Each measurement was repeated three times.

## Results

### miR396-resistant variants with in-frame *osgrf4* mediated by the CRISPR/Cas9 system

**Fig. 1.**
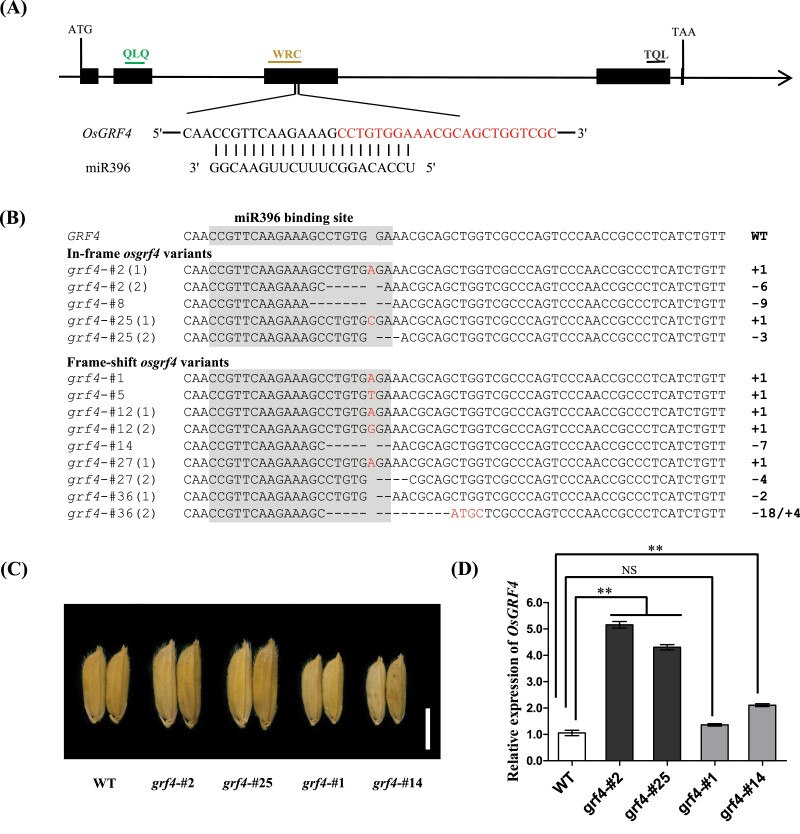
Development of large grains through CRISPR/Cas9-mediated miR396-resistant mutants of the *OsGRF4* allele. (A) Gene structure of *OsGRF4* in ‘S143’ (WT) and the miR396 target site. The sgRNA targeting the flanking sequences of the recognition site is indicated by red letters. The miR396 recognition sequence is marked in grey background. (B) Mutations in T_0_ plants harbouring in-frame *osgrf4* and frame-shift *osgrf4* variants. The introduced deletions and insertions are indicated by black dashes and red letters, respectively. Numbers on the right side indicate the lengths of indels compared with WT. –: deletion; +: insertion; combined mutations are distinguished by ‘/’. (C) Grain morphology of T_0_ mutant plants and the corresponding WT. Scale bar: 0.5 cm. (D) Expression of *OsGRF4* in young panicles of T_0_ mutant plants. Data are means ± SD. **P < 0.01 compared with WT using Student’s *t*-test. NS: no significant difference.

We picked an elite rice variety, *indica* ‘Shuhui143’ (‘S143’) to obtain miRNA-resistant mutants. Sequence analysis revealed that *OsGRF4* of ‘S143’ contained the miR396-complementary sequence of 21 nucleotides in the third exon (Fig. 1A; Supplementary Fig. S1A), To interfere with miR396-mediated repression, a Cas9/sgRNA construct targeting the flanking sequences of miR396 recognition site was designed (Fig. 1A). Through *Agrobacterium*-mediated genetic transformation, a total of 43 T_0_ independent transgenic plants were generated from calli of ‘S143’. For T_0_ lines, 26 out of 43 were identified as mutants, of which more than half (53.85%) were putatively homozygous, while heterozygous and bi-allelic lines accounted for 11.54% and 34.62% of the total mutants, respectively (Table 1; Supplementary Table S2). All of the T_0_ mutants destroyed the miR396-complementary sequence of *OsGRF4*, which would get rid of miR396-mediated post-transcriptional regulation.

**Table 1.** Summary of mutation frequencies at targeted miR396-complementary sequence of *OsGRF4* allele in T_0_ lines

No. of transgenic plants in ‘S143’	No. of plants with mutation: number, ratio^a^	T_0_ zygosity^b^			No. of plants with in-frame osgrf4 alleles: number, ratio^b^
		Homozygous: number, ratio	Heterozygous: number, ratio	Bi-allelic: number, ratio	
43	26, 60.47%	14, 53.85%	3, 11.54%	9, 34.62%	3, 11.54%

^a^ Percentages were calculated over the total number of transgenic plants.

^b^ Percentages were calculated over the total number of plants with mutations.

We aimed to screen in-frame mutations in which the number of insertion or deletion nucleotides were in multiples of three. Among the T_0_ mutants, three plants that contained an in-frame *osgrf4* allele were identified (Fig. 1B; Table 1). Compared with the WT OsGRF4 protein, the in-frame mutants (*grf4*-#2, *grf4*-#8, *grf4*-#25) had minor deletions ranging from 1–3 amino acids (Supplementary Fig. S2). These results indicated the possibility that miR396-resistant mutants with in-frame *osgrf4* might have been successfully obtained by the CRISPR/Cas9 system.

### Putative off-target analysis and T_0_ rice plants of in-frame *osgrf4* mutants exhibit increased grain size phenotype

The miR396-resistant plants were evaluated for potential off-target mutations. We examined five potential off-target sites carrying two to four mismatched bases retrieved by the online tool CRISPR-P (http://skl.scau.edu.cn/offtarget/). To further confirm whether the target sgRNA affected *OsMIR396* and other family genes additionally, both the *OsMIR396* members and other *OsGRF* genes were also detected (Supplementary Table S3). Then the PCR products that were amplified from the *osgrf4* mutants were sequenced (all primers are listed in Supplementary Table S1), and no mutation events were found across all potential off-target sites, *OsMIR396* and other *OsGRF* genes (Supplementary Table S3), showing that the *OsGRF4*-targeting sgRNA had high specificity in inducing miR396-resistant variants.

In T_0_ mutants harbouring in-frame *osgrf4* variants (*grf4*-#2, *grf4*-#25), the grain shape was larger than that of ‘S143’ WT (Fig. 1C). The corresponding *OsGRF4* transcripts were obviously elevated (Fig. 1D), indicating that *OsGRF4* was protected from the cleavage of miR396, and the function was preserved. Similarly, the expression of *OsGRF4* in the frame-shift mutant *grf4*-#14 was increased. There was no significant difference (*P>0.05*) in the *OsGRF4* transcripts between WT and *grf4*-#1, which may be because the 1 bp insertion did not affect the regulation of miR396. However, the frame-shift *osgrf4* mutants produced inactivated OsGRF4 and thus exhibited smaller grain traits (Fig. 1C, D; Supplementary Fig. S3A). To evaluate the effects of miR396 on other target genes after the de-repression of *OsGRF4* and the expression of *OsMIR396* members, we tested the transcripts of *OsGRFs and OsMIR396* genes (Supplementary Fig. S4A, B). The results showed that there were no significant differences (*P>0.05*) in the expression of other target genes and *OsMIR396* members between WT and in-frame mutants (Supplementary Fig. S4C, D; Fig. S3B). These findings corroborated that our method could make the specific target gene escape from miRNA suppression, thereby effectively improving particular traits.

### Inheritance of miR396-resistant variants analysis in T_1_ generation

**Fig. 2.**
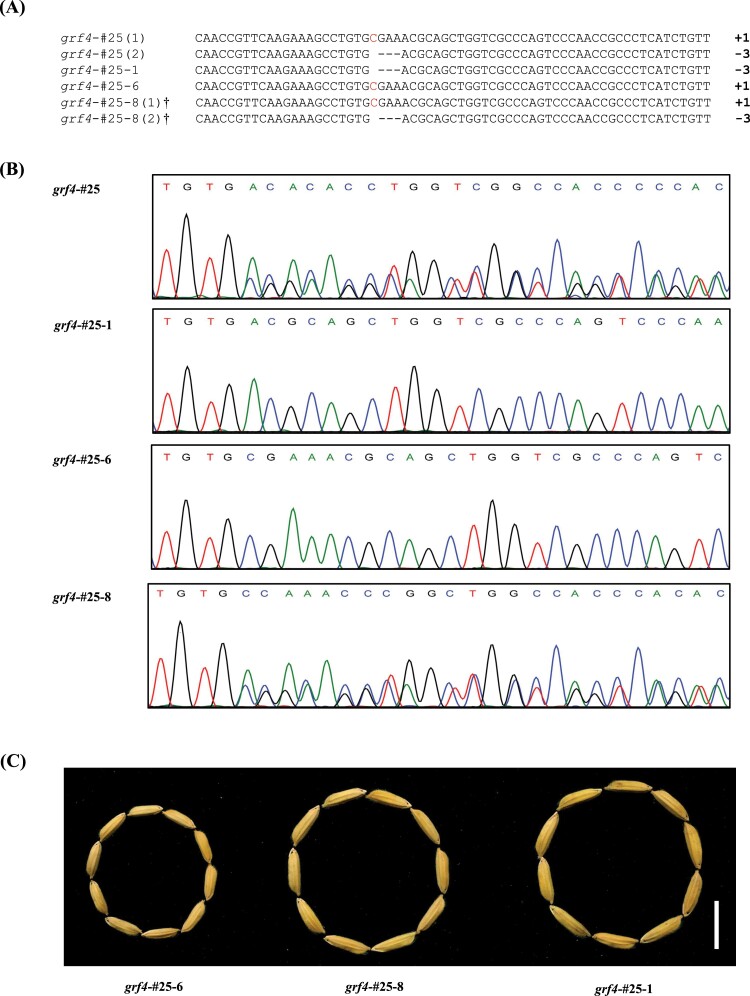
Segregation analysis of T_1_ progeny of in-fame *grf4*-#25 mutant. (A) Alignment of sequences of the indels site in T_0_ and T_1_ progeny of *grf4*-#25. †Bi-allelic sequences were decoded from sequencing chromatograms using an online program DSDecode (http://dsdecode.scgene.com/). (B) Examples of sequencing chromatograms from different mutants presented in (A). (C) Grain phenotypes of segregations in T_1_ progeny of *grf4*-#25. Scale bar: 1 cm.

To investigate the pattern of transmission of miR396-resistant mutants and segregation of the Cas9/sgRNA T-DNA, several T_1_ progeny were obtained by strict self-separation and used for testing, including in-frame variants (*grf4*-#2, *grf4*-#25) and frame-shift mutants (*grf4*-#1, *grf4*-#14). In all T_1_ progeny of frame-shift variants, both the homozygous mutant *grf4*-#1 and the heterozygous mutant *grf4*-#14 showed the same phenotypes as that of respective T_0_ generations (Table 2). However, seeds of the T_1_ bi-allele in-frame mutant plant *grf4*-#25 showed the same large-grain phenotype as the T_0_ plant; besides, some T_1_ progeny displayed small-grain traits due to the isolation of the +1 bp genotype (Fig. 2). These data indicated that the mutations in the T_0_ lines were stably transferred to the T_1_ generation and the phenotypes of grain size were consistent with the corresponding mutations. In addition, the identification of the Cas9/sgRNA T-DNA by PCR amplification determined the presence or absence of the transgene. The results showed that the T-DNA region could be segregated out in most T_1_ lines. Moreover, the transgene-free plants with in-frame *osgrf4* mutants were observed in both T_1_ progenies of *grf4*-#2 and *grf4*-#25 (Table 2).

**Table 2.** Segregation patterns of the introduced mutations during the T_0_ to T_1_ generation

Line	T_0_		T_1_			
	Zygosity^a^	Genotype^b^	No. of plants tested	No. of plants with mutation phenotype^c^	*Cas9* positive: *Cas9* negative	No. of transgene-free plants with in-frame osgrf4 allele
*grf4*-#2	Bi-allele	+1,-6	24	5(+1); 12(+1,-6); 7(-6)	13:11	9
*grf4*-#25	Bi-allele	+1,-3	24	7(+1); 11(+1,-3); 6(-3)	15:9	6
*grf4*-#1	Homozygote	+1	24	24(+1)	18:6	0
*grf4*-#14	Heterozygote	-7	24	6(WT); 12(-7,WT); 6(-7)	19:5	0

^a^ The zygosity of homozygote, heterozygote and bi-allele in T_0_ plant lines were putative.

^b^ The numbers indicate the lengths of indels compared with *OsGRF4* gene of wild type. +: insertion; -: deletion;,: Bi-allelic mutations^.^

^c^ The numbers in brackets indicate the lengths of insertions or deletions compared with *OsGRF4* gene of wild type.

### The analyses of yield traits for T_2_ progeny of miR396-resistant mutant plants

**Fig. 3.**
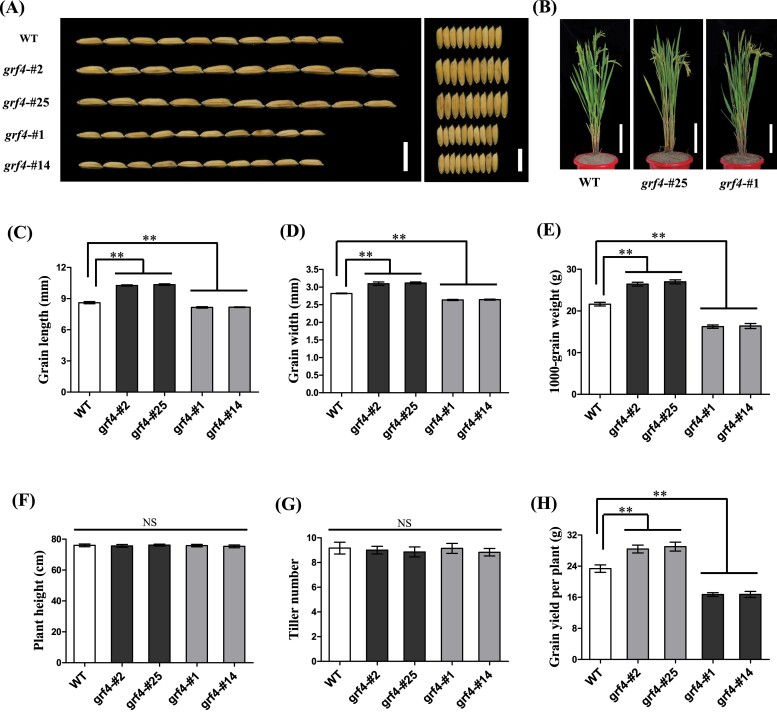
Phenotypes and grain yield analysis of T_2_ generation mutants. (A) Grain morphology of *osgrf4* mutants and the wild type ‘S143’ (WT). Scale bar: 1 cm. (B) Morphological phenotypes of *osgrf4* mutants and the corresponding WT. Scale bar: 20 cm. (C-G) Statistical data of the grain length (C), grain width (D), 1000-grain weight (E), plant height (F), till number (G), and grain yield per plant (H) of the WT and *osgrf4* variants. Values are means ± SD. **P < 0.01 compared with WT using Student’s *t*-test. NS: no significant difference.

To further examine the characteristics of miR396-resistant mutations in later generations, the phenotypes of T_2_ plants were analysed in detail. The transgene-free homozygous mutants for the *OsGRF4* were selected to investigate the yield-associated traits (Fig. 3). Consistent with the roles of the *OsGRF4* allele in regulating many important agronomic traits as reported previously (Duan *et al.*, 2015; Hu *et al.*, 2015; Che *et al.*, 2016; Chen *et al.*, 2019), *grf4-*#2 and *grf4*-#25 carrying in-frame *osgrf4* had a significantly enlarged (*P<0.01*) grain volume that was 20.21% longer and 10.56% wider than WT (Fig. 3A, C, D), resulting in a remarkable increase (23.08%) of the 1000-grain weight (Fig. 3E). In contrast, the grain size of the frame-shift *osgrf4* mutants, *grf4*-#1 and *grf4*-#14, was obviously smaller and lighter than that of WT plants (Fig. 3A, C-E). As demonstrated by scanning electron microscopy of the grain husk, the volume and number of outer epidermal cells in the in-frame mutants were enlarged and increased compared with the WT (Supplementary Fig. S5). In contrast, the cell size and cell number of the frame-shift variants were significantly reduced (*P<0.01*) in the outer surface of glumes (Supplementary Fig. S5). Results showed that in-frame *osgrf4* could remarkably increase grain size by promoting cell elongation and cell proliferation(Supplementary Fig. S5), which confirmed that *OsGRF4* positively regulated cell size and cell number to enlarge grain size (Hu *et al.*, 2015).

Gross morphologies of the *osgrf4* mutants were similar to WT plants (Fig. 3B). No significant differences (*P>0.05*) in plant height and tiller number were observed among the WT, in-frame and frame-shift mutants (Fig. 3F, G). Furthermore, to investigate the actual grain yield, we used 24 plants in the corresponding lines. As shown in Supplementary Fig. S6, the in-frame *osgrf4* panicle length were significantly longer (*P<0.05*) than WT. By contrast, branch number and grain number per panicle of in-frame *osgrf4* mutants were similar to those of WT plants. Thus, the in-frame *osgrf4* variants were notably increased by 24.15% in the grain yield per plant (Fig. 3H). Conversely, the yield per plant of frame-shift mutants was significantly reduced (*P<0.01*) because obvious changes had taken place in the yield-associated traits (Fig. 3H; Supplementary Fig. S6). Overall, we could effectively change the regulation of *OsGRF4* on grain size and rice yield by editing the miR396 recognition site, so that it would be a feasible method for rice genetic breeding.

### Application of the de-regulation of *OsGRF4* in rice breeding

To evaluate the applicability of this strategy in genetic improvement of grain shape, 30 rice accessions were collected and sequenced for the miR396 recognition site of *OsGRF4* allele. However, none of these rice cultivars contained the base substitutions (Supplementary Fig. S7), implying that our strategy had a great potential to take advantage of *OsGRF4* allele in developing the high-yield elite varieties. Notably, the sgRNA of *OsGRF4* gene designed in this study was applicable to most rice varieties (Supplementary Fig. S7), which would meet the requirements of employing the CRISPR/Cas9 system to specifically de-repress the *OsGRF4* gene and improve grain size in the interested cultivars.

### BPH resistance increased in the miR396-resistant line with in-frame *osgrf8* variants

**Fig. 4.**
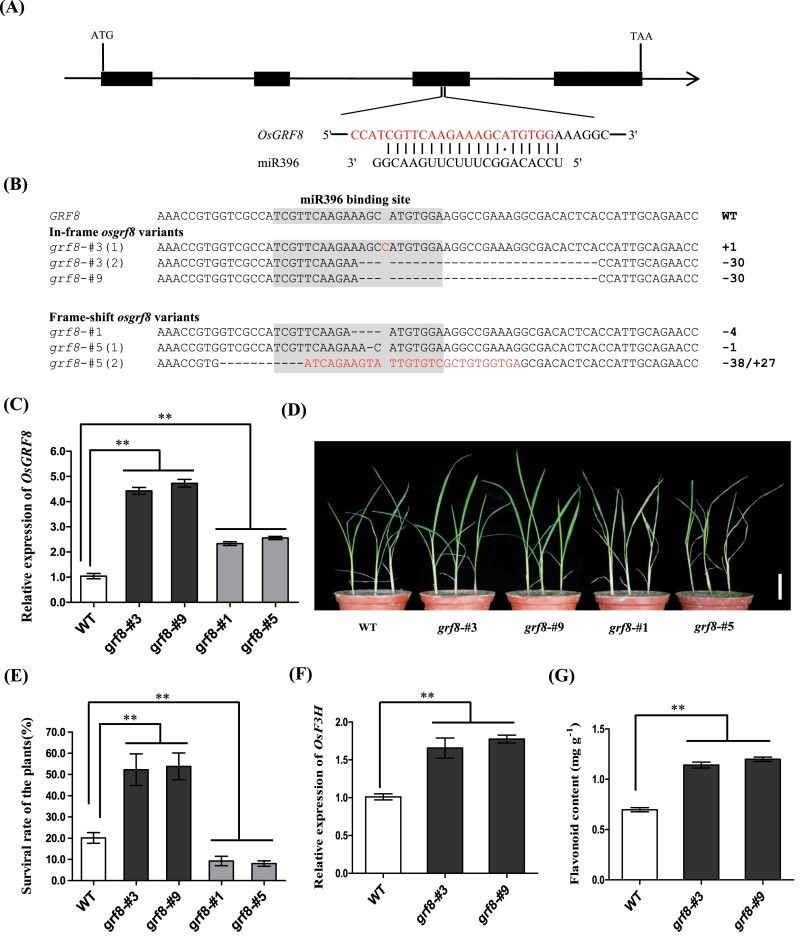
Development of BPH resistance rice through CRISPR/Cas9-mediated miR396-resistant mutants of the *OsGRF8* allele. (A) Gene structure of *OsGRF8* in ‘S143’ (WT) and the miR396 target site. The sgRNA targeting the flanking sequences of the recognition site is indicated by red letters. (B) Mutations in T_0_ plants harbouring in-frame *Osgrf8* and frame-shift *Osgrf8* variants. The miR396 recognition sequence was marked in grey background. The introduced deletions and insertions are indicated by black dashes and red letters, respectively. Numbers on the right side indicate the lengths of indels compared with WT. –: deletion; +: insertion; combined mutations are distinguished by ‘/’. (C) Expression of *OsGRF8* in leaves of T_0_ mutant plants compared with WT. (D) Individual tests to determine the BPH resistance of the WT, in-frame variants and frame-shift mutants. Scale bar: 5 cm. (E) Statistical analysis of the survival rates of WT plants and *Osgrf8* mutants after BPH feeding (*n*=3). (F) Expression of *OsF3H* in leaves of WT and in-frame *Osgrf8* plants. (G) Quantitative determination of flavonoid content in the WT and in-frame *Osgrf8* mutants (*n*=3). Data are mean ± SD. **P<0.01 compared with WT using Student’s *t*-test.

To verify the feasibility of this strategy, we selected another functional gene inhibited by miRNA for further investigations. Previous studies reported that miR396 regulated *OsGRF8* to affect brown planthopper (BPH) resistance (Dai *et al.*, 2019). Consequently, a sgRNA targeting the flanking sequences of the recognition site in the *OsGRF8* allele was designed to circumvent miR396 suppression (Fig. 4A; Supplementary Fig. S1B). Both the in-frame and frame-shift mutants disrupted the miR396 recognition sequence and escaped the inhibition by miR396 (Fig. 4B). Hence, *OsGRF8* transcripts were significantly elevated (*P<0.01*) in the mutant plants (Fig. 4C; Supplementary Fig. S3C). However, OsGRF8 in the frame-shift mutants were inactivated and lost their normal functions. In addition, to investigate the specificity of the *OsGRF8* target site, the *OsMIR396* genes and other *OsGRF* family alleles were selected (Supplementary Table S4). We found that all *osgrf8* variants had no mutations in the *OsMIR396* and other *OsGRF* genes, probably because the fourth base of the sgRNA was unique, resulting in a high specificity of the *OsGRF8* target site.

To evaluate the response to BPH, the T_2_ generation of *osgrf8* mutants and the WT ‘S143’ were selected for testing. After BPH infestation for 7 d, the in-frame mutants *grf8*-#3 and *grf8*-#9 showed greater resistance to BPH than the WT, and accordingly, the survival rate increased (Fig. 4D, E). On the contrary, the frame-shift lines *grf8*-#1 and *grf8*-#5 were the most sensitive to brown planthopper (Fig. 4D, E). Subsequently, the expression of *OsF3H* activated by OsGRF8 was detected in the WT and in-frame plants, which positively correlated with flavonoid content and BPH resistance. As expected, the expression of *OsF3H* was elevated in the in-frame variants (Fig. 4F; Supplementary Fig. S3D). Correspondingly, the increased flavonoid content was also found in the in-frame plants (Fig. 4G). These findings confirmed that the strategy could introduce the miR396-resistant mutants in rice and improve BPH resistance in the in-frame mutants.

## Discussion

Plant miRNAs have recently presented as promising targets for crop improvement, and the target mimicry and STTM have been developed to inhibit the activity of miRNAs. However, neither of the MIM or STTM approaches guarantees the strongest silencing efficacies of miRNA activity (Liang *et al.*, 2014; Reichel *et al.*, 2015). The 1 bp mutations mediated by CRISPR/Cas9 are a little difficult to block the suppression of miRNA on the target genes, due to the fact that the base-frame shift may not impact the process of miRNA maturation, and may generate the functionally redundant miRNAs (Zhang *et al.*, 2021; Zhou *et al.*, 2017). Meanwhile, these miRNA-target technologies probably produce the compounded phenotypes when a miRNA possesses multiple target genes (Zhang *et al.*, 2017). To precisely promote the expression of specific target genes regulated by miRNA, we make in-frame mutations on the miRNA recognition site, and circumvent miRNA suppression successfully. Given a list of studies reported that altering the expression of target genes repressed by miRNAs could significantly affect grain yield (Zhao *et al.*, 2017; Jiang *et al.*, 2018; Zhao *et al.*, 2019), and change stress responses (Dai *et al.*, 2019), it is conceivable that our strategy may be promising to accurately improve elite traits in crop breeding.

The strategy appears to be simple and precise for improving specific traits. However, we have to take into consideration whether the deletions of amino acids in the miRNA binding region disrupt the protein function. If so, this strategy will not work. OsGRF4 belongs to the GRF transcription factor family, and possesses conserved QLQ, WRC and TQL domains (Li *et al.*, 2016). The in-frame mutants, *grf4*-#2, *grf4*-#8 and *grf4*-#25, has amino acids missing ranging from 1–3 in the C-terminal of the WRC domain (Supplementary Fig. S2). However, the deletions avoided destroying the CCCH zinc-finger motif, which preserves the function of the WRC domain. Because the conserved QLQ, WRC and TQL domains were maintained (Supplementary Fig. S2), the in-frame variants still retained the regulation of OsGRF4 on the grain size. Therefore, the in-frame variants could precisely manipulate agronomic traits regulated by miRNAs, on the premise that the major domains were retained.

Previous studies reported that *GS*^*AA*^, which was substituted at the miR396 recognition site of *OsGRF4*, increased the grain size (Duan *et al.*, 2015; Hu *et al.*, 2015; Che *et al.*, 2016; Chen *et al.*, 2019). But the in-frame *osgrf4* mutants we obtained had amino acid deletions. In order to identify whether the emerged large-grain phenotype was caused by the newly generated protein function or by miR396 de-regulation, we designed a target site sgRNA-2 on the miR396-complementary sequence of *OsGRF4* (Supplementary Fig. S8A). The in-frame mutants, *grf4-2*-#7 and *grf4-2*-#46, which had lost amino acids 12 and 7 respectively, produced different OsGRF4 proteins (Supplementary Fig. S8B, C). However, the T_1_ progeny of *grf4-2*-#7 and *grf4-2*-#46 still displayed a large-grain phenotype, consistent with the traits of *grf4-*#2 and *grf4*-#25 (Supplementary Fig. S8D, E). These results indicated that the large-grain phenotype of in-frame *osgrf4* mutants were caused by getting rid of miR396 inhibition rather than the new protein functions.

The miRNA-resistant mutants with in-frame targets relieve the influence of endogenous miRNA regulation, elevating the expression of corresponding target genes, and manipulating agronomic traits precisely. Most of the knockout mutants mediated by CRISPR/Cas9 system usually produce 1 bp indels between the third and fourth bases upstream of the Protospacer Adjacent Motif (PAM ) region (Zhang *et al.*, 2014). Whereas, the acquisition rate of mutants with 3n bp indels is relatively low. To increase the number of in-frame mutants, we can introduce two adjacent sgRNAs simultaneously whose intervals between PAM sequences is 3n bp. Thereby, the likelihood of the missing bases harbouring a multiple of three would increase (Guo *et al.*, 2018). Similar to obtaining miRNA-resistant targets, the improved base editing and prime editing technologies can be designed to replace the miRNA recognition sequence of the target gene, which seem to have more potential (Kim *et al.*, 2017; Anzalone *et al.*, 2019). However, the unsatisfactory editing efficiency (Butt *et al.*, 2020; Xu *et al.*, 2020) and the off-target editing effects of cytosine base editor have limited their applications in crop breeding presently (Jin *et al.*, 2019). Thus, employing CRISPR/Cas9 system to accurately develop specific traits inhibited by miRNAs is promising.

In summary, CRISPR/Cas9-mediated destruction of miR396 inhibition elevated the *OsGRF4* and *OsGRF8* transcripts in the corresponding in-frame variants, leading to the enlarged grain size and increased BPH resistance. These results proved that our strategy could be effectively employed to precise de-regulation of target genes repressed by miRNAs and would be of great significance for production of elite traits in plants.

## Supplementary data

The following supplementary data are available at *JXB* online.

Fig. S1. Sequences of the third exon of the *OsGRF4* and *OsGRF8* alleles in ‘S143’.

Fig. S2. Multiple alignment of the deduced amino acid sequences of in-frame *osgrf4* mutants.

Fig. S3. qRT–PCR analysis of a second reference gene *UBIQUITIN*.

Fig. S4. Analysis of the expression of miR396 target genes and *OsMIR396* members.

Fig. S5. Scanning electron microscopy observation of spikelet lemma.

Fig. S6. Analyses of panicle traits for wild type ‘S143’ and *osgrf4* mutants.

Fig. S7. Alignment of the miR396 complementary site in *OsGRF4* from rice conventional varieties.

Fig. S8. Development of large grain through the sgRNA-2 of the *OsGRF4* allele.

Table S1. Primers used in this study.

Table S2. Genotypes of T_0_ mutant plants in ‘S143’.

Table S3. Evaluation of sgRNA-*OsGRF4* potential off-target sites.

Table S4. Evaluation of sgRNA-*OsGRF8* potential off-target sites.

erab336_suppl_Supplementary_Figures_Tables

## Data Availability

All data supporting the findings of this study are available within the paper and within its supplementary materials published online.
