# Supplementary material for: Derepression of specific miRNA-target genes in rice using CRISPR/Cas9
Source: J Exp Bot. 2021 Jul 20;72(20):7067–77. doi: 10.1093/jxb/erab336 (PMC8547147; doi:10.1093/jxb/erab336)
Supplement: erab336_suppl_Supplementary_Figures_Tables [file erab336_suppl_supplementary_figures_tables.pdf]

Supplemental Information

Derepression of specific miRNA-target genes in rice using CRISPR/Cas9

Contents

Supplemental Materials and Methods

Fig. S1. Sequences of the third exon of the *OsGRF4* and *OsGRF8* alleles in ‘S143’.

Fig. S2. Multiple alignment of the deduced amino acid sequences of in-frame *osgrf4* mutants.

Fig. S3. qRT–PCR analysis of a second reference gene *UBIQUITIN*.

Fig. S4. Analysis of the expression levels of miR396 target genes and *OsMIR396* members.

Fig. S5. Scanning electron microscopic observation of spikelet lemma.

Fig. S6. Analyses of panicle traits for wild type ‘S143’ and *osgrf4* mutants.

Fig. S7. Alignment of the miR396 complementary site in *OsGRF4* from rice conventional varieties.

Fig. S8. Development of large grain through the sgRNA-2 of the *OsGRF4* allele.

Table S1. Primers used in this study.

Table S2. Genotypes of T_0 mutant plants in ‘S143’.

Table S3. Evaluation of sgRNA-*OsGRF4* potential off-target sites.

Table S4. Evaluation of sgRNA-*OsGRF8* potential off-target site.

(A)

TTGGATATGGTCCGTACTTCGGCAAGAAGCTGGACCCAGAGCCAGGGCGGTGCCGGCGTA

CGGACGGCAAGAAATGGCGGTGCTCGAAGGAGGCCGCGCCGGATTCCAAGTACTGCGAGC

GCCACATGCACCGCGGCCGCAACCGTTCAAGAAAGCCTGTGGAAACGCAGCTGGTCGCCC

AGTCCCAACCGCCCTCATCTGTTGTCGGTTCTGCGGCGGCGCCCCTTGCTGCTGCCTCCA

ATGGCAGCAGCTTCCAAAACCACTCTCTTTACCCTGCTATTGCCGGCAGCAATGGCGGGG

GCGGGGGGAGGAACATGCCCAGCTCATTTGGCTCGGCGTTGGGTTCTCAGCTGCACATGG

ATAATGCTGCCCCTTATGCAGCTGTTGGTGGTGGAACAGGCAAAGATCTCAG

(B)

GATGGGGATCATTCCCTCCTGGCTGTGCTGATGTAGAACCCAGAAGATGCCGCCGCACAG

ACGGCAAGAAGTGGCGGTGCTCCAGAGATGCTGTTGGGGATCAGAAGTATTGTGAGCGAC

ACATAAACCGTGGTCGCCATCGTTCAAGAAAGCATGTGGAAGGCCGAAAGGCGACACTCA

CCATTGCAGAACCATCCATGGTTATTGCTGCTGGTGTATCATCTCGCGGCCACACTGTGG

CTCGGCAGAAGCAGGTGAAAGGCTCAGCTGCTACTGTCTCTGATCCTTTCTCGAGACAAT

CCAACAG

Fig. S1. Sequences of the third exon of the *OsGRF4* and *OsGRF8* alleles in 'S143'. (A) sequence of the third exon in *OsGRF4*. (B) sequence of the third exon in *OsGRF8*. The miR396 complementary sequence of 21 nucleotides were indicated in red words.

```
WT          MAMPYASLSPAVADHRSSPAAATASLLPFCRSTPLSAGGGGVAMGEDAPMTARWPPAAAA   60
grf4-#2     MAMPYASLSPAVADHRSSPAAATASLLPFCRSTPLSAGGGGVAMGEDAPMTARWPPAAAA   60
grf4-#8     MAMPYASLSPAVADHRSSPAAATASLLPFCRSTPLSAGGGGVAMGEDAPMTARWPPAAAA   60
grf4-#25    MAMPYASLSPAVADHRSSPAAATASLLPFCRSTPLSAGGGGVAMGEDAPMTARWPPAAAA   60

                           QLQ
WT          RLPPFTAAQYEELEQQALIYKYLVAGVPVPPDLVLPIRRGLDSLAARFYNHPALGYGPYF  120
grf4-#2     RLPPFTAAQYEELEQQALIYKYLVAGVPVPPDLVLPIRRGLDSLAARFYNHPALGYGPYF  120
grf4-#8     RLPPFTAAQYEELEQQALIYKYLVAGVPVPPDLVLPIRRGLDSLAARFYNHPALGYGPYF  120
grf4-#25    RLPPFTAAQYEELEQQALIYKYLVAGVPVPPDLVLPIRRGLDSLAARFYNHPALGYGPYF  120

                                   WRC
WT          GKKLDPEPGRCRRTDGKKWRCSKEAAPDSKYCERHMHRGRNRSRKPVETQLVAQSQPPSS  180
grf4-#2     GKKLDPEPGRCRRTDGKKWRCSKEAAPDSKYCERHMHRGRNRSRK--QTQLVAQSQPPSS  180
grf4-#8     GKKLDPEPGRCRRTDGKKWRCSKEAAPDSKYCERHMHRGRNRSSP---TQLVAQSQPPSS  180
grf4-#25    GKKLDPEPGRCRRTDGKKWRCSKEAAPDSKYCERHMHRGRNRSRKPV-TQLVAQSQPPSS  180

WT          VVGSAAAPLAAASNGSSFQNHSLYPAIAGSNGGGGGRNMPSSFGSALGSQLHMDNAAPYA  240
grf4-#2     VVGSAAAPLAAASNGSSFQNHSLYPAIAGSNGGGGGRNMPSSFGSALGSQLHMDNAAPYA  240
grf4-#8     VVGSAAAPLAAASNGSSFQNHSLYPAIAGSNGGGGGRNMPSSFGSALGSQLHMDNAAPYA  240
grf4-#25    VVGSAAAPLAAASNGSSFQNHSLYPAIAGSNGGGGGRNMPSSFGSALGSQLHMDNAAPYA  240

WT          AVGGGTGKDLRYTAYGTRSLADEQSQLITEAINTSIENPWRLLPSQNSPFPLSSYSQLGA  300
grf4-#2     AVGGGTGKDLRYTAYGTRSLADEQSQLITEAINTSIENPWRLLPSQNSPFPLSSYSQLGA  300
grf4-#8     AVGGGTGKDLRYTAYGTRSLADEQSQLITEAINTSIENPWRLLPSQNSPFPLSSYSQLGA  300
grf4-#25    AVGGGTGKDLRYTAYGTRSLADEQSQLITEAINTSIENPWRLLPSQNSPFPLSSYSQLGA  300

WT          LSDLGQNTPSSLSKVQRQPLSFFGNDYAAVDSVKQENQTLRPFFDEWPKGRDSWSDLADE  360
grf4-#2     LSDLGQNTPSSLSKVQRQPLSFFGNDYAAVDSVKQENQTLRPFFDEWPKGRDSWSDLADE  360
grf4-#8     LSDLGQNTPSSLSKVQRQPLSFFGNDYAAVDSVKQENQTLRPFFDEWPKGRDSWSDLADE  360
grf4-#25    LSDLGQNTPSSLSKVQRQPLSFFGNDYAAVDSVKQENQTLRPFFDEWPKGRDSWSDLADE  360

                       TQL
WT          NANLSSFSGTQLSISIPMASSDFSAASSRSTNGD*   395
grf4-#2     NANLSSFSGTQLSISIPMASSDFSAASSRSTNGD*   395
grf4-#8     NANLSSFSGTQLSISIPMASSDFSAASSRSTNGD*   395
grf4-#25    NANLSSFSGTQLSISIPMASSDFSAASSRSTNGD*   395
```

Fig. S2. Multiple alignment of the deduced amino acid sequences of in-frame *osgrf4* mutants. The QLQ domain is boxed in green, the WRC domain in yellow, and the TQL domain in gray background. In the WRC domain, CCCH-type zinc finger motif is indicated by red letters.

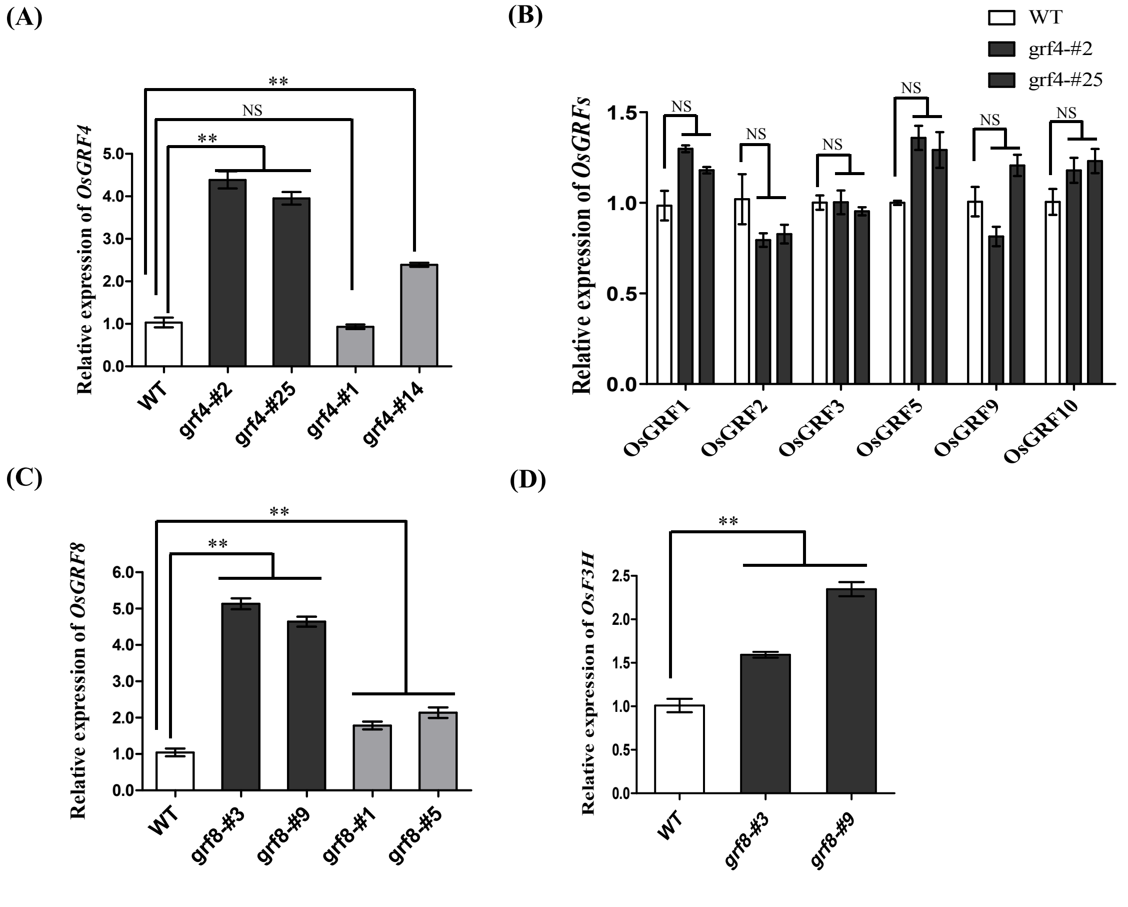

Fig. S3. qRT–PCR analysis of a second reference gene *UBIQUITIN*. (A) Expression of *OsGRF4* in young panicles of T_0 mutant plants. (B) Expression of *OsGRF* genes in young panicles of in-frame *osgrf4* plants. (C) Expression of *OsGRF8* in leaves of T_0 mutant plants compared with 'S143' (WT). (D) Expression of *OsF3H* in leaves of the WT and in-frame *osgrf8* plants. Data are presented as mean ± SD. ***P*<0.01 compared with WT using Student's *t*-test. NS: no significant difference.

(A)

```
OsMIR396     3' GGCAAGUUCUUUCGGACACCU 5'
                ||||||||||||||||||||| 
  OsGRF1     5' CCGTTCAAGAAAGCCTGTGGA 3'
  OsGRF2     5' CCGTTCAAGAAAGCCTGTGGA 3'
  OsGRF3     5' CCGTTCAAGAAAGCCTGTGGA 3'
  OsGRF4     5' CCGTTCAAGAAAGCCTGTGGA 3'
  OsGRF5     5' CCGTTCAAGAAAGCCTGTGGA 3'
  OsGRF9     5' CCGTTCAAGAAAGCCTGTGGA 3'
  OsGRF10    5' CCGTTCAAGAAAGCCTGTGGA 3'
```

(B)

```
OsMIR396a    3' GUCAAGUUCUUUCG·ACACCUU    5'
OsMIR396b    3' GUCAAGUUCUUUCG·ACACCUU    5'
OsMIR396c    3' UUCAAGUUCUUUCG·ACACCUU    5'
OsMIR396d    3' GGCAAGUUCUUUCGGACACCU     5'
OsMIR396e    3' GUCAAGUUCUUUCGGACACCU     5'
OsMIR396f    3'  UCAAGUUCUUUCGGACACCUCU   5'
OsMIR396g    3' GGCAAGUUCUUUCGGACACCU     5'
OsMIR396h    3' GGCAAGUUCUUUCGGACACCU     5'
                 |||||||||||||||||||||
OsGRF4       5' CCGTTCAAGAAAGCCTGTGGA     3'
```

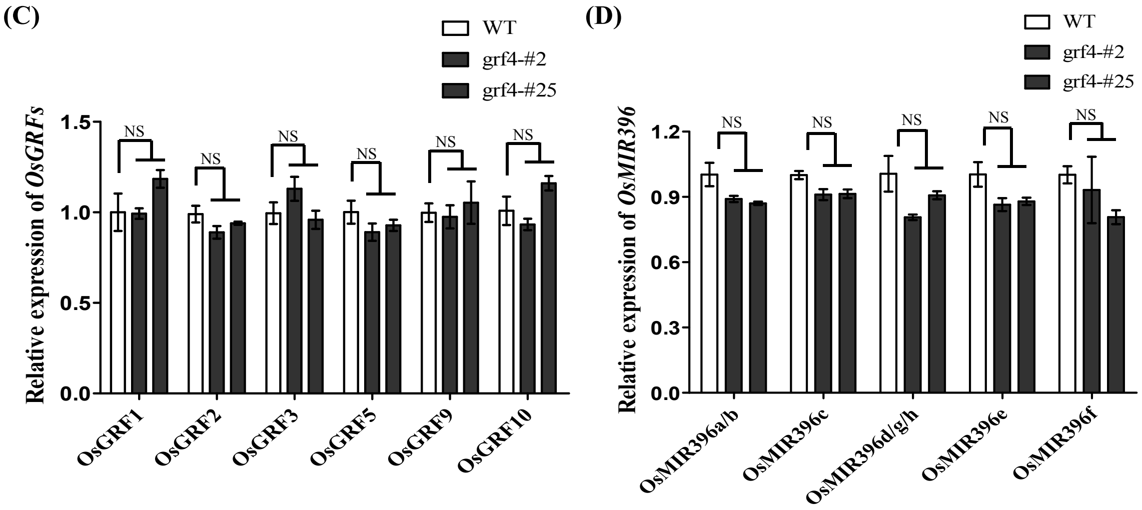

Fig. S4. Analysis of the expression of miR396 target genes and *OsMIR396* members. (A) Sequence alignment of *OsmiR396d* mature sequences with complementary sequences of *OsGRF* genes. (B) Pairing of the *OsGRF4* gene by *OsMIR396* members. The sequences that are perfectly complementary to the *OsGRF4* gene are shown in red. (C) Expression of *OsGRF* genes in young panicles of in-frame *osgrf4* plants (n=3). (D) qRT–PCR analysis of *OsMIR396* expression in young panicles of in-frame *osgrf4* plants (n=3). Values are given as the mean ± SD. NS: no significant difference.

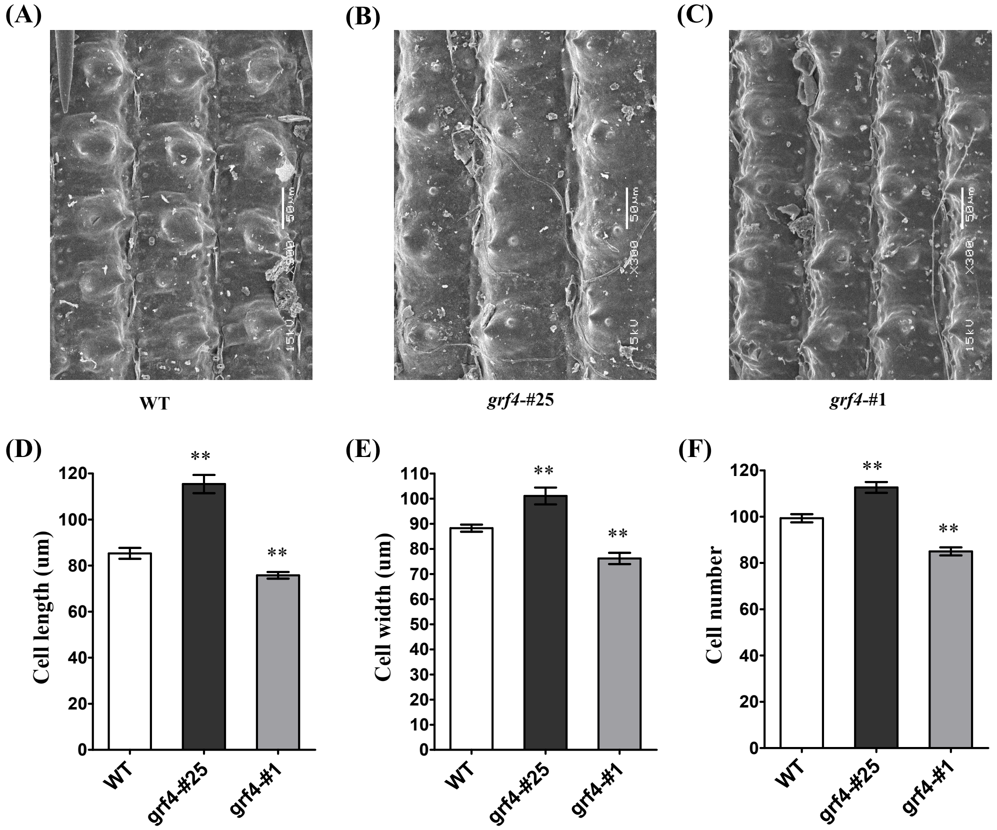

Fig. S5. Scanning electron microscopy observation of spikelet lemma. (A-C) Outer epidermal cells of the 'S143' (WT) (A), *grf4*-#25 (B) and *grf4*-#1 (C) lemmas. Scale bar, 50 µm. (D, E) Comparison analysis of cell length and width in outer glumes. (F) The calculated number of outer epidermal cells of WT and *osgrf4* mutants lemmas in the longitudinal direction. Values are means ± SD. ***P*<0.01 compared with WT using Student's *t*-test.

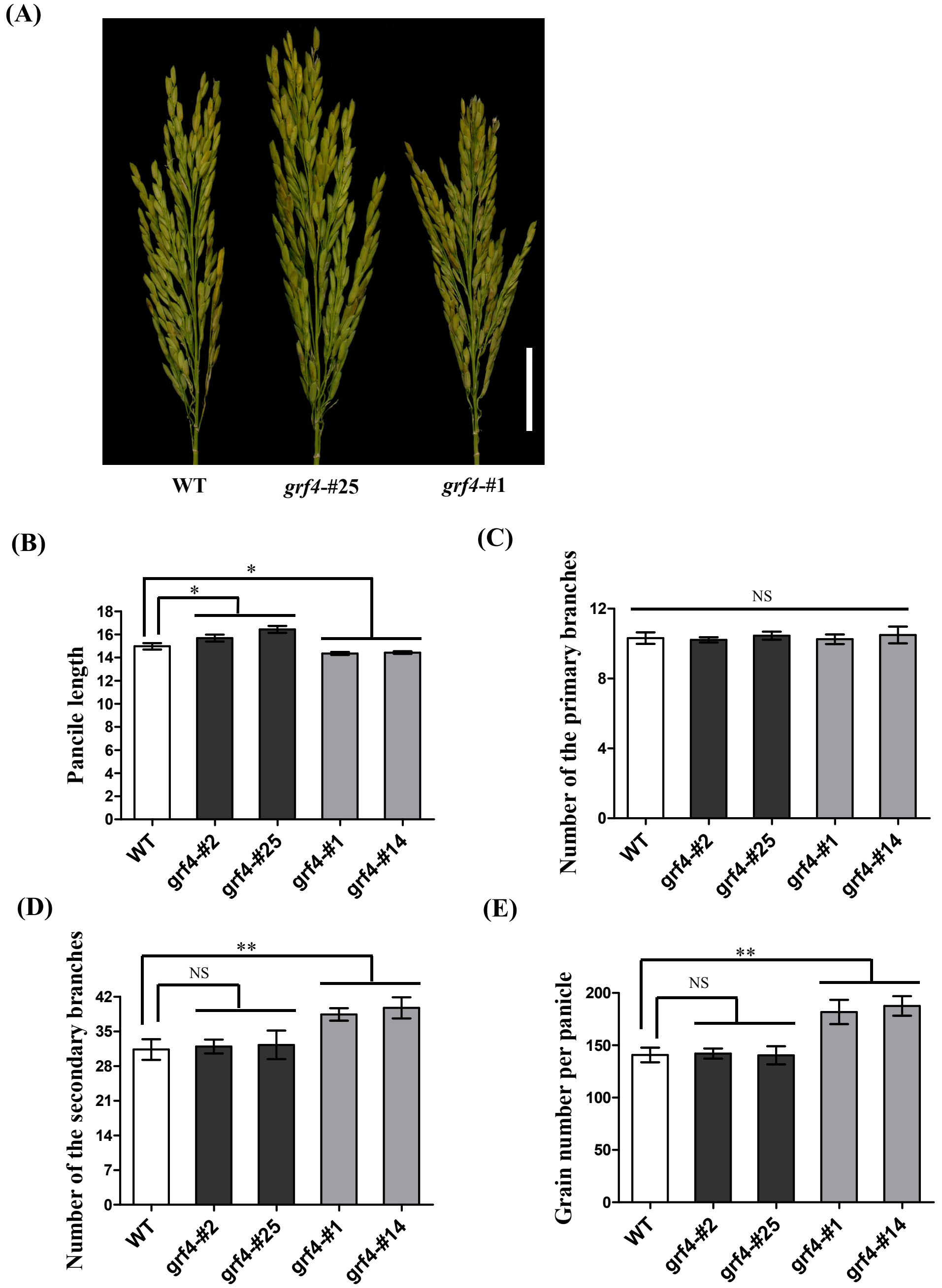

Fig. S6. Analyses of panicle traits for wild type ‘S143’ and *osgrf4* mutants. (A) Comparison of the panicle morphologies among the ‘S143’ (WT) , in-frame and frame-shift *osgrf4* mutants. Scale bar: 3 cm. (B-E) panicle length (B), number of primary branches (C), number of secondary branches (D), and grain number per panicle (E) of WT and *osgrf4* mutants. Over 36 replicates were analyzed for panicle length, primary branch number, secondary branch number and grain number. Values are means ± SD. *P<0.05 and **P<0.01 compared with WT using Student’s t-test. NS: no significant difference.

```
3' GCAAGUUCUUUCGGACACCUU 5'   OsmiR396
   |||||||||||||||||||||
   CGTTCAAGAAAGCCTGTGGAAACGCAGCTGGTCGC    Shuhui143
   CGTAAAAGAAAGCCTGTGGAAACGCAGCTGGTCGC    Judali
   ***  ******************************
   CGTTCAAGAAAGCCTGTGGAAACGCAGCTGGTCGC    Nipponbare
   CGTTCAAGAAAGCCTGTGGAAACGCAGCTGGTCGC    Xiushui 134
   CGTTCAAGAAAGCCTGTGGAAACGCAGCTGGTCGC    Shuhui 527
   CGTTCAAGAAAGCCTGTGGAAACGCAGCTGGTCGC    Bobai B
   CGTTCAAGAAAGCCTGTGGAAACGCAGCTGGTCGC    Zhehui 911
   CGTTCAAGAAAGCCTGTGGAAACGCAGCTGGTCGC    Guanghui 128
   CGTTCAAGAAAGCCTGTGGAAACGCAGCTGGTCGC    Jigeng 91
   CGTTCAAGAAAGCCTGTGGAAACGCAGCTGGTCGC    Teqing 2
   CGTTCAAGAAAGCCTGTGGAAACGCAGCTGGTCGC    Jinghui 20
   CGTTCAAGAAAGCCTGTGGAAACGCAGCTGGTCGC    Shenhui 3
   CGTTCAAGAAAGCCTGTGGAAACGCAGCTGGTCGC    Shenhui 6
   CGTTCAAGAAAGCCTGTGGAAACGCAGCTGGTCGC    Xiaolihui 8
   CGTTCAAGAAAGCCTGTGGAAACGCAGCTGGTCGC    Gu 154
   CGTTCAAGAAAGCCTGTGGAAACGCAGCTGGTCGC    Zhonghua 11
   CGTTCAAGAAAGCCTGTGGAAACGCAGCTGGTCGC    Yanxigengnuo
   CGTTCAAGAAAGCCTGTGGAAACGCAGCTGGTCGC    Dongtingwanxian
   CGTTCAAGAAAGCCTGTGGAAACGCAGCTGGTCGC    Changlihui
   CGTTCAAGAAAGCCTGTGGAAACGCAGCTGGTCGC    ITA 233
   CGTTCAAGAAAGCCTGTGGAAACGCAGCTGGTCGC    PT 193
   CGTTCAAGAAAGCCTGTGGAAACGCAGCTGGTCGC    PT 278
   CGTTCAAGAAAGCCTGTGGAAACGCAGCTGGTCGC    PT 332
   CGTTCAAGAAAGCCTGTGGAAACGCAGCTGGTCGC    PT 359
   CGTTCAAGAAAGCCTGTGGAAACGCAGCTGGTCGC    PT 382
   CGTTCAAGAAAGCCTGTGGAAACGCAGCTGGTCGC    J 1
   CGTTCAAGAAAGCCTGTGGAAACGCAGCTGGTCGC    J 13
   CGTTCAAGAAAGCCTGTGGAAACGCAGCTGGTCGC    J 21
   CGTTCAAGAAAGCCTGTGGAAACGCAGCTGGTCGC    J 22
   CGTTCAAGAAAGCCTGTGGAAACGCAGCTGGTCGC    J 25
   CGTTCAAGAAAGCCTGTGGAAACGCAGCTGGTCGC    D 8110
   CGTTCAAGAAAGCCTGTGGAAACGCAGCTGGTCGC    G 9138
```

Fig. S7. Alignment of the miR396 complementary site in *OsGRF4* from rice conventional varieties. Green letters indicate the mutation site in *OsGRF4* mRNA of large grain variety Judali, red letters indicate the sgRNA of *OsGRF4* gene in this study.

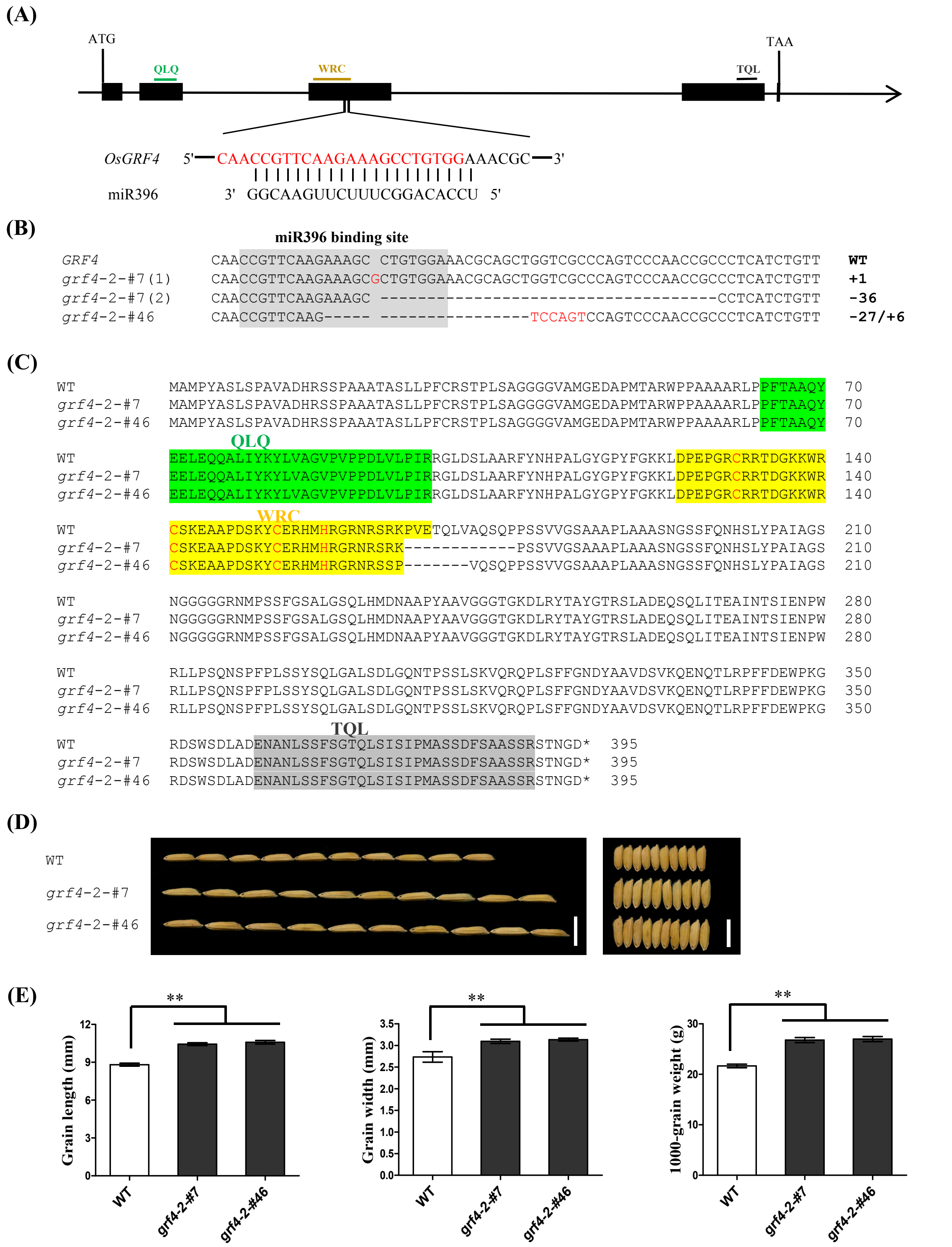

Fig. S8. Development of large grain through the sgRNA-2 of the *OsGRF4* allele. (A) Gene structure of *OsGRF4* in 'S143' and the miR396 target site. The sgRNA-2 targeting the flanking sequences of the recognition site is indicated by red letters. (B) Mutations in T_0 plants in-frame *osgrf4* variants. The miR396 recognition sequence was marked in gray background. The introduced deletions and insertions are indicated by black dashes and red letters, respectively. Numbers on the right side indicate the lengths of indels compared with wild type. –: deletion; +: insertion; combined mutations are distinguished by '/'. (C) Multiple alignment of the deduced amino acid sequences of in-frame *osgrf4* mutants. The QLQ domain is boxed in green, the WRC domain in yellow, and the TQL domain in gray background. In the WRC domain, CCCH-type zinc finger motif is indicated by red letters. (D) Grain morphology of T_1 in-frame mutant plants and the corresponding wild type. Scale bar: 1 cm. (E) Statistical data of the grain length, grain width and 1000-grain weight. Data are presented as mean ± SD. $^{**}P<0.01$ compared with wild type using Student's *t*-test.

Table S1. Primers used in this study

Primers	Forward/Reverse sequence (5'-3') †	Comment
sgRNA-GRF4	F: CAGGCGACCAGCTGCGTTTCCAC<br>R: AACGTGGAAACGCAGCTGGTCGC	Primers for making Cas9/sgRNA construct
sgRNA-2-GRF4	F: CAGGAACCGTTCAAGAAAGCCTG<br>R: AACCAGGCTTTCTTGAACGGTTC	Primers for making Cas9/sgRNA construct
sgRNA-GRF8	F: CAGCCATCGTTCAAGAAAGCATG<br>R: AACCATGCTTTCTTGAACGATGG	Primers for making Cas9/sgRNA construct
Cas9 gene	F: GGGAGATCCAGCTAGAGGTC<br>R: GGAAGGAGGAAGACAAGG	Primers for examining the transgenic plants
GRF4-exon3	F: TGAGCGATTGCTAAGAACA<br>R: TAGACAACGGAAGGATACATA	Primers for examining the miR396d recognized sequence in S143
GRF8-exon3	F: CAGACCTGGGAGGAATGTT<br>R: AGGTTGTTAGGATTTGGCAC	Primers for examining the miR396a/b recognized sequence in S143
qRT-PCR-GRF1	F: CTCCTGGATGGGGCTCG<br>R: TCATCATTGTGGTAGCGGGA	Primers for detecting the expression of *OsGRF4* in the young panicles
qRT-PCR-GRF2	F: TTCCCTCCCCATCACCTG<br>R: CTCCACCCTCCTCCCGTA	Primers for detecting the expression of *OsGRF4* in the young panicles

Table S1. Primers used in this study (Continued)

Primers	Forward/Reverse sequence (5’-3’) †	Comment
qRT-PCR-GRF3	F: CTTCGGGAGCGACTATGTGA<br>R: GTGGCAAGGCTGTTGTCATC	Primers for detecting the expression of *OsGRF4* in the young panicles
qRT-PCR-GRF4	F: ACGGCAAGAAATGGCGGT<br>R: CCGCAGAACCGACAACAGAT	Primers for detecting the expression of *OsGRF4* in the young panicles
qRT-PCR-GRF5	F: TCGCCTCGCTGTCCCAAG<br>R: TCATCCATCTCCGACCACGA	Primers for detecting the expression of *OsGRF4* in the young panicles
qRT-PCR-GRF8	F: AGATGCCGCCGCACAGA<br>R: GCAATGGTGAGTGTCGCCTT	Primers for detecting the expression of *OsGRF8* in the leaves
qRT-PCR-GRF9	F: TCGTGGATGCCTTCAGTCAGT<br>R: CTCTGGCGAGGAATAGAAAGTTG	Primers for detecting the expression of *OsGRF4* in the young panicles
qRT-PCR-GRF10	F: CCGTTCAAGAAAGCCTGTGG<br>R: ACGCTGGTGGGGGAGAAG	Primers for detecting the expression of *OsGRF4* in the young panicles
OsActin1	F: TCTGTATGCCAGTGGTCGT<br>R: GCCGTTGTGGTGAATGAG	Primers as an endogenous control for qRT-PCR
qRT-PCR-F3H	F: AGCAACGGAAGATACAAGAGCG<br>R: GCACAGGAACGATGCGACG	Primers for detecting the expression of *OsF3H* in the *OsGRF8* mutants

Table S1. Primers used in this study (Continued)

Primers	Forward/Reverse sequence (5'-3') †	Comment
OFF1	F: CGTTGACTCCCATAGACAGC	Primers for PCR amplification of the potential off-target sites sgRNA-*OsGRF4* in transgenic plants
	R: ATTGAAGCACGTGGCCTT	
OFF2 (GRF3)	F: TTGCGTGCTTGGTTGGTCTG	Primers for PCR amplification of the potential off-target sites sgRNA-*OsGRF4* and sgRNA-*OsGRF8* in transgenic plants
	R: CCGCCATTAGCAATCGCC	
OFF3	F: TGGGATGGAATGCGGTCT	Primers for PCR amplification of the potential off-target sites sgRNA-*OsGRF4* in transgenic plants
	R: GAGGCGGCAGATAAGGTTTT	
OFF4	F: CGAGAGGGAAATGTTTGAGA	Primers for PCR amplification of the potential off-target sites sgRNA-*OsGRF4* in transgenic plants
	R: CAGCAAAAGTGAGAACTACCC	
OFF5	F: GTGCGTCTTGCCGTGGTT	Primers for PCR amplification of the potential off-target sites sgRNA-*OsGRF4* in transgenic plants
	R: CGTCGTCTTCCTGCTCCCT	
GRF1	F: TGGGGTGGGGTTGCTTTG	Primers for PCR amplification of the potential off-target sites sgRNA-*OsGRF4* and sgRNA-*OsGRF8* in transgenic plants
	R: GGCGTGGCCAAGGACATT	
GRF2	F: CGTTCCGATCGATGCGTA	Primers for PCR amplification of the potential off-target sites sgRNA-*OsGRF4* and sgRNA-*OsGRF8* in transgenic plants
	R: TGCTCCTTGGACAACGACC	
GRF4	F: GTTGCTACGATGTGCCTGTT	Primers for PCR amplification of the potential off-target sites sgRNA-*OsGRF8* in transgenic plants
	R: CGGCAATAGCAGGGTAAAG	

Table S1. Primers used in this study (Continued)

Primers	Forward/Reverse sequence (5'-3')[†]	Comment
GRF5	F: ATTCAATGTGCCTGTGCTCA<br>R: GCACCACCAAGGGACAGAC	Primers for PCR amplification of the potential off-target sites sgRNA-*OsGRF4* and sgRNA-*OsGRF8* in transgenic plants
GRF6	F: TGGTTGGGTTTTTGCTTTG<br>R: GCAGTGTTGGCAGCGTAGT	Primers for PCR amplification of the potential off-target sites sgRNA-*OsGRF4* and sgRNA-*OsGRF8* in transgenic plants
GRF7	F: TTACAGTGGGATGGGGGT<br>R: TTAGTAGCCAAGGTTTTCGT	Primers for PCR amplification of the potential off-target sites sgRNA-*OsGRF4* and sgRNA-*OsGRF8* in transgenic plants
GRF8	F: GTTGCTACGATGTGCCTGTT<br>R: CGGCAATAGCAGGGTAAAG	Primers for PCR amplification of the potential off-target sites sgRNA-*OsGRF4* in transgenic plants
GRF9	F: ATCAAGGCTTCCCAAATGC<br>R: TGCGTGGCTTCATTTCATTA	Primers for PCR amplification of the potential off-target sites sgRNA-*OsGRF4* and sgRNA-*OsGRF8* in transgenic plants
GRF10	F: ATGTGGACGCCGTTTTTG<br>R: CTGCGGATTTGGGTTTTC	Primers for PCR amplification of the potential off-target sites sgRNA-*OsGRF4* and sgRNA-*OsGRF8* in transgenic plants
GRF12	F: TGTCGTTGGTCATTCCGTGT<br>R: TGGGCGAGGAGCACTTTG	Primers for PCR amplification of the potential off-target sites sgRNA-*OsGRF4* and sgRNA-*OsGRF8* in transgenic plants
OsMIR396a	F: AACCTCTCTCCACCAACC<br>R: AACTGCTCTCTCTGATGCC	Primers for PCR amplification of the potential off-target sites sgRNA-*OsGRF8* in mutants

Table S1. Primers used in this study (Continued)

Primers	Forward/Reverse sequence (5'-3') †	Comment
OsMIR396b	F: AACCTCTCACCAACCCCAT<br>R: GGTACCTTGCATCTACATCCTAA	Primers for PCR amplification of the potential off-target sites sgRNA-*OsGRF8* in transgenic plants
OsMIR396c	F: GGAGAGGTGTTGCAATGTG<br>R: CTCTCTGCCTGTCTCATGG	Primers for PCR amplification of the potential off-target sites sgRNA-*OsGRF8* in transgenic plants
OsMIR396d	F: TGATGAGTGGGCTGTGCT<br>R: CTTGGATACGGTTCCTACTTC	Primers for PCR amplification of the potential off-target sites sgRNA-*OsGRF4* and sgRNA-*OsGRF8* in transgenic plants
OsMIR396e	F: AGAGCAGGACACCGTACTA<br>R: TGAACCATCAACAGGTAGAG	Primers for PCR amplification of the potential off-target sites sgRNA-*OsGRF4* and sgRNA-*OsGRF8* in transgenic plants
OsMIR396f	F: CTCCTCTTCTTCGCATCGT<br>R: CACCATGTTTTCCAAGGACT	Primers for PCR amplification of the potential off-target sites sgRNA-*OsGRF4* in transgenic plants
OsMIR396g	F: GAAGGAGTCGAGGTGGAGCTG<br>R: TGGGGTGGGGGTGCTATG	Primers for PCR amplification of the potential off-target sites sgRNA-*OsGRF4* and sgRNA-*OsGRF8* in transgenic plants
OsMIR396h	F: GCGTGGCCAAGGACATTT<br>R: GCACCAGGCGCTCATCTAC	Primers for PCR amplification of the potential off-target sites sgRNA-*OsGRF4* and sgRNA-*OsGRF8* in transgenic plants
Ubiquitin	F: GAAGAAGTGTGGTCACAGCAAC<br>R: GATAACAACGGAAGCATAAAAGTC	Primers as an endogenous control for qRT-PCR analysis

Table S1. Primers used in this study (Continued)

Primers	Forward/Reverse sequence (5'-3') †	Comment
MIR396a/b/e-RT	GTCGTATCCAGTGCAGGGTCCGAGGTATT CGCACTGGATACGACCAGTTC	Primer for miR396 qRT-PCR analysis
MIR396c-RT	GTCGTATCCAGTGCAGGGTCCGAGGTATT CGCACTGGATACGACAAGTTC	Primer for miR396 qRT-PCR analysis
MIR396d/g/h-RT	GTCGTATCCAGTGCAGGGTCCGAGGTATT CGCACTGGATACGACCCGTTC	Primer for miR396 qRT-PCR analysis
MIR396f-RT	GTCGTATCCAGTGCAGGGTCCGAGGTATT CGCACTGGATACGACAGTTCA	Primer for miR396 qRT-PCR analysis
qRT-MIR396a/b/c F	CGCGTTCCACAGCTTTCTT	Primer for miR396 qRT-PCR analysis
qRT-MIR396d/g/h/e F	CGCGTCCACAGGCTTTCTT	Primer for miR396 qRT-PCR analysis
qRT-MIR396f F	CGCGTCTCCACAGGCTTTCT	Primer for miR396 qRT-PCR analysis
qRT-MIR396 R	AGTGCAGGGTCCGAGGTATT	Primer for miR396 qRT-PCR analysis
qRT-PCR-U6	F: TACAGATAAGATTAGCATGGCCCC<br>R: GGACCATTTCTCGATTTGTACGTG	Primers as an endogenous control for miR396 qRT-PCR analysis

†The primers of forward and reverse are represented by 'F', 'R' respectively.

Table S2. Genotypes of T_0 mutant plants in 'S143'

T_0 plant of grf4	Mutation sequences†	Length of deletion/insertion (bp)‡	Zygosity
#1	CGTTCAAGAAAGCCTGTGAGAAACGCAGCTGGTCGCCCAGT	+1	Homozygous
#2	CGTTCAAGAAAGCCTGTGAGAAACGCAGCTGGTCGCCCAGT	+1	Bi-allelic
	CGTTCAAGAAAGC----- -AAACGCAGCTGGTCGCCCAGT	-6	
#4	CGTTCAAGAAAGCCTGTG -AAACGCAGCTGGTCGCCCAGT	-1	Homozygous
#5	CGTTCAAGAAAGCCTGTGTGAAACGCAGCTGGTCGCCCAGT	+1	Homozygous
#7	CGTTCAAGAAAGCCTGTGAGAAACGCAGCTGGTCGCCCAGT	+1	Homozygous
#8	CGTTCAAGAAA------- --AACGCAGCTGGTCGCCCAGT	-9	Heterozygous
#9	CGTTCAAGAAAGCCTGTGTGAAACGCAGCTGGTCGCCCAGT	+1	Heterozygous
#11	CGTTCAAGAAAGCCTGTGAGAAACGCAGCTGGTCGCCCAGT	+1	Homozygous
#12	CGTTCAAGAAAGCCTGTGAGAAACGCAGCTGGTCGCCCAGT	+1	Bi-allelic
	CGTTCAAGAAAGCCTGTGGGAAACGCAGCTGGTCGCCCAGT	+1	
#13	CGTTCAAGAAAGCCTGTGAGAAACGCAGCTGGTCGCCCAGT	+1	Homozygous
#14	CGTTCAAGAAAGC----- --AACGCAGCTGGTCGCCCAGT	-7	Heterozygous
#16	CGTTCAAGAAAGCCTGTGTGAAACGCAGCTGGTCGCCCAGT	+1	Bi-allelic
	CGTTCAAGAAAGCCTGTG ----CGCAGCTGGTCGCCCAGT	-4	
#17	CGTTCAAGAAAGCCTGTGTGAAACGCAGCTGGTCGCCCAGT	+1	Homozygous
#19	CGTTCAAGAAAGCCTGTGAGAAACGCAGCTGGTCGCCCAGT	+1	Homozygous

Table S2. Genotypes of T_0 mutant plants in 'S143' (Continued)

T_0 plant of grf4	Mutation sequences[†]	Length of deletion/insertion (bp)[‡]	Zygosity
#20	CGTTCAAGAAAGCCTGTGTGAAACGCAGCTGGTCGCCCAGT	+1	Bi-allelic
	CGTTCAAGAAAGCCTGTG --AACGCAGCTGGTCGCCCAGT	-2	
#25	CGTTCAAGAAAGCCTGTGCGAAACGCAGCTGGTCGCCCAGT	+1	Bi-allelic
	CGTTCAAGAAAGCCTGTG ---ACGCAGCTGGTCGCCCAGT	-3	
#27	CGTTCAAGAAAGCCTGTGAGAAACGCAGCTGGTCGCCCAGT	+1	Bi-allelic
	CGTTCAAGAAAGCCTGTG ----CGCAGCTGGTCGCCCAGT	-4	
#28	CGTTCAAGAAAGCCTGTGGGAAACGCAGCTGGTCGCCCAGT	+1	Homozygous
#29	CGTTCAAGAAAGCCTGTGTGAAACGCAGCTGGTCGCCCAGT	+1	Homozygous
#30	CGTTCAAGAAAGCCTGTGTGAAACGCAGCTGGTCGCCCAGT	+1	Homozygous
#35	CGTTCAAGAAAGCCTGTGAGAAACGCAGCTGGTCGCCCAGT	+1	Homozygous
#36	CGTTCAAGAAAGCCTGTG --AACGCAGCTGGTCGCCCAGT	-2	Bi-allelic
	CGTTCAAGAAAGC----- ---------ATGCTCGCCCAGT	-18/+4	
#37	CGTTCAAGAAAGCCTGTGAGAAACGCAGCTGGTCGCCCAGT	+1	Homozygous
#38	CGTTCAAGAAAGCCTGTGTGAAACGCAGCTGGTCGCCCAGT	+1	Bi-allelic
	CGTTCAAGAAAGCCTGTG ----CGCAGCTGGTCGCCCAGT	-4	
#42	CGTTCAAGAAAGCCTGTGAGAAACGCAGCTGGTCGCCCAGT	+1	Bi-allelic
	CGTTCAAGAAAGCCTGTGCGAAACGCAGCTGGTCGCCCAGT	+1	
#43	CGTTCAAGAAAGCCTGTGAGAAACGCAGCTGGTCGCCCAGT	+1	Homozygous

[†]Newly introduced deletions and insertions are respectively indicated by black dashes and red letters.

[‡]The numbers indicated the lengths of deletions or insertion compared with wild type in S143; -: deletion; +: insertion; combined mutations are distinguished by '/'.

Table S3. Evaluation of sgRNA-*OsGRF4* potential off-target sites

Name of putative off-target sites	Putative off-target locus	Putative off-target sequence†	No. of mismatch bases	No. of indel mutation
OFF1	chr03: 8069930-8069952	TCGTCCAGCTGCGTTGCCACCGG	3	0
OFF2 (GRF3)	chr04: 30320171-30320193	GCGACGAGCTGCGCTTCCACAGG	2	0
OFF3	chr06: 26779377-26779399	GGGACCAGCTGCACTTCAACAGG	4	0
OFF4	chr02: 30824711-30824733	GCGACAGGCTGCGCCTCCACAGG	4	0
OFF5	chr09: 15897531-15897553	GCCACCAGCTGCGCGTCCACCGG	3	0
GRF1	chr02: 32841756-32841778	GTGGCCAAGGACATTTCCACAGG	7	0
GRF2	chr06: 5300653-5300675	GCGGGGGGCGGCATTTCCACAGG	6	0
GRF5	chr06: 895279-895301	GCAGCGGTCTTGGATTCCACAGG	8	0
GRF6	chr03: 29824026-29824048	TGGCCAGGCTGGCCTTCCACATG	9	0
GRF7	chr12: 17917715-17917737	TGGCTAGATTGGCCTTCCACATG	12	0
GRF8	chr11: 20523905-20523927	GTCGCCTTTCGGCCTTCCACATG	10	0
GRF9	chr03: 26680877-26680899	TTTCTACTTTGGTTTTCCACAGG	11	0
GRF10	chr02: 27728708-27728730	GCGGCCGCGGAGGCTTCCACAGG	8	0
GRF12	chr04: 28923008-28923030	ACTGCTGCAGAGGCTTCCATAGG	12	0
OsMIR396d	chr04: 30320171-30320193	GCGACGAGCTGCGCTTCCACAGG	2	0
OsMIR396e	chr04: 34436817-34436839	TGTGCGGGCATGCTTTCCACAGG	10	0

Table S3. Evaluation of sgRNA-*OsGRF4* potential off-target sites (Continued)

Name of putative off-target sites	Putative off-target locus	Putative off-target sequence†	No. of mismatch bases	No. of indel mutation
OsMIR396f	chr02: 35636705-35636727	ATTGCGGCCATGCTCTCCACAGG	12	0
OsMIR396g	chr06: 5300653-5300675	GCGGGGGGCGGCATTTCCACAGG	6	0
OsMIR396h	chr02: 32841756-32841778	GTGGCCAAGGACATTTCCACAGG	7	0

†The blue letters indicate PAM sequence NGG. Mismatch nucleotides are marked in red.

Table S4. Evaluation of sgRNA-*OsGRF8* potential off-target sites

Name of putative off-target sites	Putative off-target locus	Putative off-target sequence†	No. of mismatch bases	No. of indel mutation
OFF1 (GRF7)	chr12: 17917699-17917721	CCACCGTTCAAGAAAGCATGTGG	1	0
OFF2 (GRF6)	chr03: 29824010-29824032	CCACCGTTCAAGAAAGCATGTGG	1	0
OFF3 (GRF9)	chr03: 26680861-26680883	CCACCGTTCAAGAAAGCCTGTGG	2	0
OFF4 (GRF3)	chr04: 30320187-30320209	CAACCGTTCAAGAAAGCCTGTGG	3	0
OFF5 (GRF4)	chr02: 28865709-28865731	CAACCGTTCAAGAAAGCCTGTGG	3	0
OFF6 (GRF5)	chr06: 895295-895317	CAACCGTTCAAGAAAGCCTGTGG	3	0
OFF8 (GRF2)	chr06: 5300637-5300659	GAACCGTTCAAGAAAGCCTGTGG	4	0
OFF9 (GRF1)	chr02: 32841772-32841794	GAACCGTTCAAGAAAGCCTGTGG	4	0
OFF10 (GRF10)	chr02: 27728692-27728714	CGGCCGTTCAAGAAAGCCTGTGG	4	0
OFF11 (GRF12)	chr04: 28922992-28923014	CGGCCGTTCAAGAAAGCCTATGG	5	0
OsMIR396a	chr02: 34280396-34280418	CGTGCAGTTCAAGAAAGCTGTGG	12	0
OsMIR396b	chr06: 3669661-3669683	GATGCAGTTCAAGAAAGCTGTGG	13	0
OsMIR396c	chr02: 34287979-34288001	AGAGAAGTTCAAGAAAGCTGTGG	13	0
OsMIR396d	chr04: 30320187-30320209	CAACCGTTCAAGAAAGCCTGTGG	3	0
OsMIR396e	chr04: 34436833-34436855	TCACAGTTCAAGAAAGCCTGTGG	4	0
OsMIR396f	chr02: 35636689-35636711	TCACAGTTCAAGAAAGCCTGTGG	4	0

Table S4. Evaluation of sgRNA-*OsGRF8* potential off-target sites (Continued)

Name of putative off-target sites	Putative off-target locus	Putative off-target sequence†	No. of mismatch bases	No. of indel mutation
OsMIR396g	chr06: 5300637-5300659	GAACCGTTCAAGAAAGCCTGTGG	4	0
OsMIR396h	chr02: 32841772-32841794	GAACCGTTCAAGAAAGCCTGTGG	4	0

†The blue letters indicate PAM sequence NGG. Mismatch nucleotides are marked in red.
